# Advance Care Planning Among Users of a Patient Portal During the COVID-19 Pandemic: Retrospective Observational Study

**DOI:** 10.2196/21385

**Published:** 2020-08-11

**Authors:** Jennifer D Portz, Adreanne Brungardt, Prajakta Shanbhag, Elizabeth W Staton, Seuli Bose-Brill, Chen-Tan Lin, Jean S Kutner, Hillary D Lum

**Affiliations:** 1 Division of General Internal Medicine Department of Medicine University of Colorado School of Medicine Aurora, CO United States; 2 Division of Geriatric Medicine Department of Medicine University of Colorado School of Medicine Aurora, CO United States; 3 Department of Family Medicine University of Colorado School of Medicine Aurora, CO United States; 4 Department of Medicine The Ohio State University College of Medicine Columbus, OH United States

**Keywords:** advance care planning, electronic health records, pandemic, COVID-19, advance directives, patient portal, planning, web-based tool, health system

## Abstract

**Background:**

Advance care planning is the process of discussing health care treatment preferences based on patients’ personal values, and it often involves the completion of advance directives. In the first months of 2020, a novel coronavirus, severe acute respiratory syndrome coronavirus 2 (SARS-CoV-2), began circulating widely in the American state of Colorado, leading to widespread diagnosis of coronavirus disease (COVID-19), hospitalizations, and deaths. In this context, the importance of technology-based, non–face-to-face methods to conduct advance care planning via patient portals has increased.

**Objective:**

The aim of this study was to determine the rates of use of a web-based advance care planning tool through a health system–based electronic patient portal both before and in the early months of the COVID-19 pandemic.

**Methods:**

In 2017, we implemented web-based tools through the patient portal of UCHealth’s electronic health record (EHR) for patients to learn about advance care planning and complete an electronically signed medical durable power of attorney (MDPOA) to legally appoint a medical decision maker. Patients accessing the portal can complete and submit a legally valid MDPOA, which becomes part of their medical record. We collected data on the patients’ date of MDPOA completion, use of advance care planning messaging, age, sex, and geographic location during the early phase of the COVID-19 pandemic (December 29, 2019, to May 30, 2020).

**Results:**

Over a 5-month period that includes the early phase of the COVID-19 pandemic in Colorado, total monthly use of the advance care planning portal tool increased from 418 users in January to 1037 users in April and then decreased slightly to 815 users in May. The number of MDPOA forms submitted per week increased 2.4-fold after the stay-at-home order was issued in Colorado on March 26, 2020 (*P*<.001). The mean age of the advance care planning portal users was 47.7 years (SD 16.1), and 2206/3292 (67.0%) were female. Women were more likely than men to complete an MDPOA, particularly in younger age groups (*P*<.001). The primary use of the advance care planning portal tools was the completion of an MDPOA (3138/3292, 95.3%), compared to sending an electronic message (148/3292, 4.5%). Over 50% of patients who completed an MDPOA did not have a prior agent in the EHR.

**Conclusions:**

Use of a web-based patient portal to complete an MDPOA increased substantially during the first months of the COVID-19 pandemic in Colorado. There was an increase in advance care planning that corresponded with state government shelter-in-place orders as well as public health reports of increased numbers of COVID-19 cases and deaths. Patient portals are an important tool for providing advance care planning resources and documenting medical decision makers during the pandemic to ensure that medical treatment aligns with patient goals and values.

## Introduction

On March 11, 2020, the World Health Organization characterized coronavirus disease (COVID-19) as a pandemic. At that time, approximately 118,000 cases had been reported in 114 countries, and 4291 people had died worldwide due to COVID-19. As of June 2020, the fatality rates for COVID-19 have been estimated to be between 0.6% and 5%; these rates are highest among older adults and people with chronic conditions [1,2]. In the United States, the Centers for Disease Control and Prevention reports that 14% of patients with COVID-19 are hospitalized and 2% are admitted to an intensive care unit (ICU) [2]. In these cases, invasive procedures such as mechanical ventilation and extracorporeal membrane oxygenation may be used to treat seriously ill patients with COVID-19. As such, there is a need to discuss goals of care and desired treatments with patients, ideally before they become critically ill.

Advance care planning is the process of discussing medical treatment preferences based on personal values, and it often involves the completion of advance directives [3,4]. In the United States, advance directives are state-specific documents that include medical durable powers of attorney (MDPOAs) and living wills [5]. Advance care planning is associated with increased advance directive documentation, completion of medical orders for life-sustaining treatment preferences, and positive health outcomes, including reduced end-of-life hospitalizations, enhanced patient-provider communication, prevention of unwanted treatment, and improved family experience [6,7].

COVID-19 has stimulated advance care planning in emergency departments and ICUs, indicating a need to provide advance care planning interventions and resources prior to hospital admission [8]. Telehealth is the use of electronic information or telecommunication technologies to promote health and health services [9]. With increased social distancing and patient concerns about in-person clinic visits [8,10,11], telehealth use has rapidly increased. Centers for Medicare & Medicaid Services reimbursement has rapidly expanded to cover telehealth services such as nonemergent clinical visits, screening for COVID-19, and advance care planning counseling [12]. Telehealth has potential to provide health care services and applicable advance care planning tools during the pandemic and beyond [13,14]. Telehealth-based advance care planning programs are feasible to deliver and effective in improving advance care planning knowledge, communication, and advance directive documentation [15].

Patient portals provide patients with secure access to personal health information contained in their electronic health record [16]. In addition to the ability to view health information, patient portals support patient-initiated entry of health information and electronic interactions between patients and providers; this promotes engagement in care, including advance care planning [17-19]. Portals thus offer a particularly accessible platform for patients and care partners to learn about COVID-19 treatment options and communicate goals of care with their providers. Over the last 5 years, our research team has partnered with health care system information technology experts, patients, caregivers, and providers to develop, test, and implement patient portal advance care planning strategies [20-24]. Specifically, the UCHealth patient portal offers patients the ability to learn more about advance care planning through web-based resources and provides the ability to create an electronically signed MDPOA form to choose a medical decision maker. The objective of this study is to examine advance care planning patient portal usage during the early stages of the COVID-19 pandemic.

## Methods

This retrospective observational study examined advance care planning by patients using web-based advance care planning tools available through the UCHealth patient portal that enable the completion of legally valid MDPOA forms. UCHealth is a regional health care system serving Colorado (including metropolitan Denver, Colorado Springs, and Northern Colorado), southern Wyoming, and western Nebraska. In 2019, 1.9 million patients received care in 12 UCHealth hospitals and 800 outpatient clinics. My Health Connection is the UCHealth patient portal integrated within the Epic electronic health record (Epic Systems, version 2017); as of May 2020, approximately 735,000 patients had a My Health Connection account. The Colorado Multiple Institutional Review Board approved this project as an evaluation of a clinical initiative.

### Patient Portal–Based Advance Care Planning Tools

**Figure 1:**
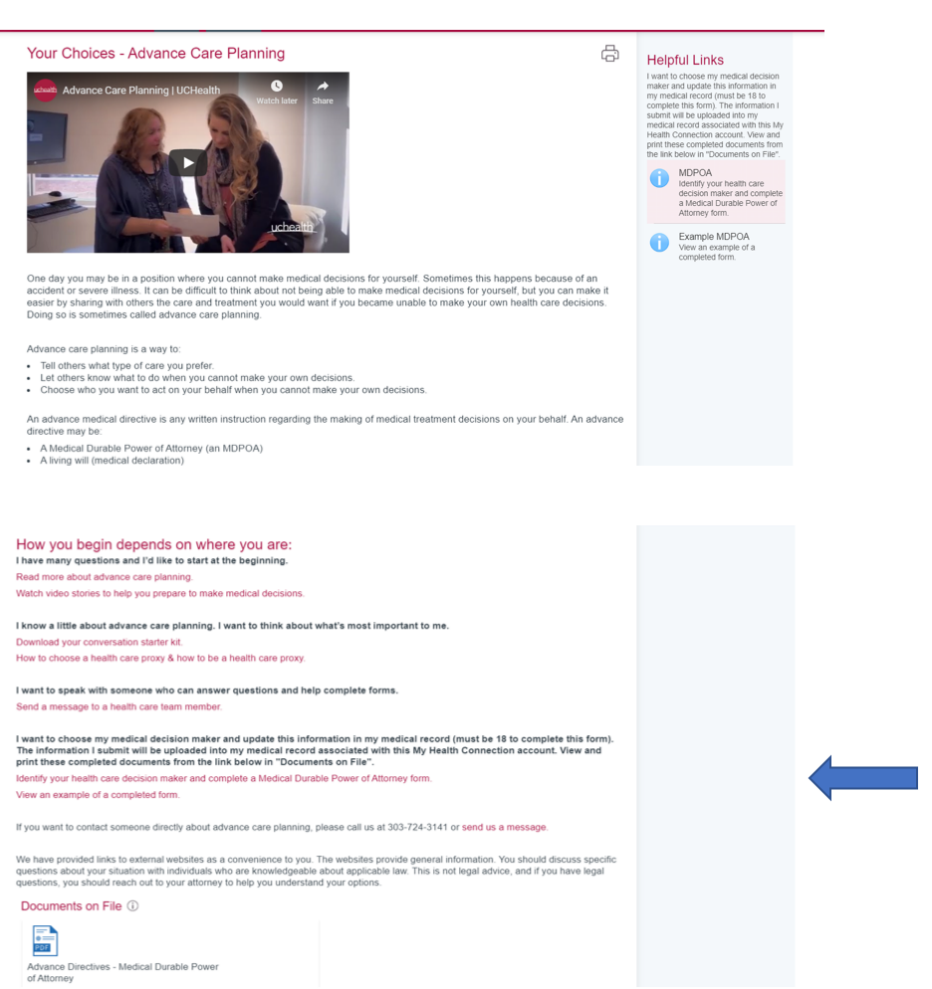
Screenshot of the advance care planning tools in the My Health Connection portal.

We previously developed and tested tools to conduct advance care planning using the My Health Connection patient portal [22,23]. Briefly, since July 2017, the advance care planning portal tools has provided patients with access to evidence-based resources, the ability to send web-based messages with questions about advance care planning, and the ability to complete a legally valid state-specific electronic MDPOA form to choose a medical decision maker. Colorado law allows patients to create an electronic MDPOA with a valid patient signature (including electronic signatures) and does not require witnesses or notary signatures. To complete the MDPOA form (Figure 1), a patient-initiated questionnaire provides information about appointing a medical decision maker, shows the exact language of the legal MDPOA form, automatically provides the name of any previously charted medical decision maker from the EHR (“orally appointed” or from a pre-existing MDPOA), and then allows the patient to name a decision maker and add up to two alternative decision makers. In this process, a printable MDPOA form is created with an electronic signature and date/time stamp. The decision maker information is displayed in a specific area of the EHR that is accessed by clinical teams. The advance care planning support team is notified when an electronic MDPOA form is submitted, briefly reviews the patient’s problem list and relevant clinical documentation for possible decision-making incapacity, then sends a message to the patient to confirm receipt of the MDPOA form. For out-of-state patients, this message notes that the MDPOA is valid for medical care received in Colorado. Patients can also use the portal message feature to contact the health system’s centralized advance care planning support team for questions and follow-up. As additional background, in Colorado, an orally appointed decision maker is a surrogate decision maker verbally chosen by an individual; however, that surrogate decision maker does not have as much legal authority as an MDPOA.

### Data Collection

Data were collected from December 29, 2019, to May 30, 2020, an approximately 5-month period that includes the 2 months prior to community spread of severe acute respiratory syndrome coronavirus 2 (SARS-CoV-2) infection in Colorado. These specific dates were chosen to enable comparison of 7-day weekly periods. The first COVID-19 diagnosis in Colorado was announced on March 5, 2020. Thus, January and February were effectively “pre–COVID-19” months in Colorado. Colorado implemented a statewide stay-at-home order on March 26, 2020. We collected data through chart review for UCHealth patients who interacted with the My Health Connection patient portal advance care planning tools, specifically by completing an electronic MDPOA form or by sending an electronic message to the advance care planning support team. Demographic information included age, sex, and geographic location (ie, metro Denver, northern Colorado, southern Colorado, or out-of-state address). To explore how patients who submitted an electronic MDPOA were choosing their health care agents, we categorized patients into the following five groups: 1) first-time designation of an MDPOA, no prior agent (orally appointed or MDPOA) in the EHR; 2) prior orally appointed agent in the EHR who is retained in the electronic MDPOA; 3) prior MDPOA on file and the same agent is retained in the electronic MDPOA; 4) prior MDPOA on file and the electronic MDPOA names a new agent; and 5) prior orally appointed agent in the EHR and the electronic MDPOA names a new agent. A prior MDPOA could include any MDPOA form in the patient’s EHR (either a paper document that was scanned into the patient’s record or a previously submitted electronic MDPOA). We also identified invalid MDPOA submissions based on advance care planning support team review and discussion with the patient or their clinical team, as previously described [23]. Invalid MDPOA submissions often included MDPOA forms submitted by someone other than the patient when the patient did not have decision-making capacity to choose a health care agent.

### Data Analysis

We used descriptive analyses to describe the characteristics of the patient portal users and expressed them as frequencies with percentages. Age categories were chosen to align with state-based surveys of community-based advance care planning rates [25]. We used chi-square tests to test gender differences across the age group distribution. Time series analyses were conducted on all data. To explore the changes in the use of the advance care planning tools in the patient portal, we used 9 weeks of data before and after March 26, 2020 (the start date of Colorado’s stay-at-home order). All tests for statistical significance were two-tailed, and a *P* value <.05 was considered statistically significant. All statistical analyses were performed using SAS version 9.4 (SAS Institute).

## Results

**Figure 2:**
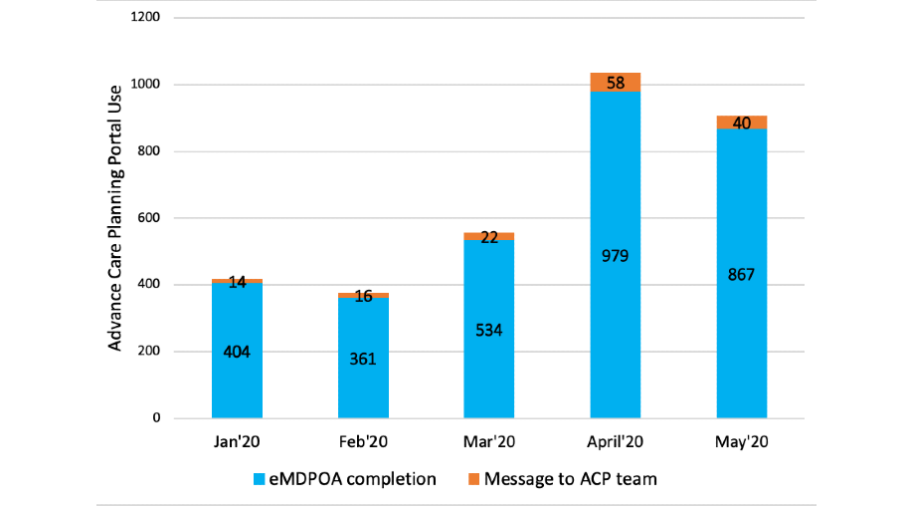
Monthly use of the UCHealth advance care planning portal during the coronavirus disease pandemic by type of portal use. ACP: advance care planning; eMDPOA: electronic medical durable power of attorney.

**Figure 3:**
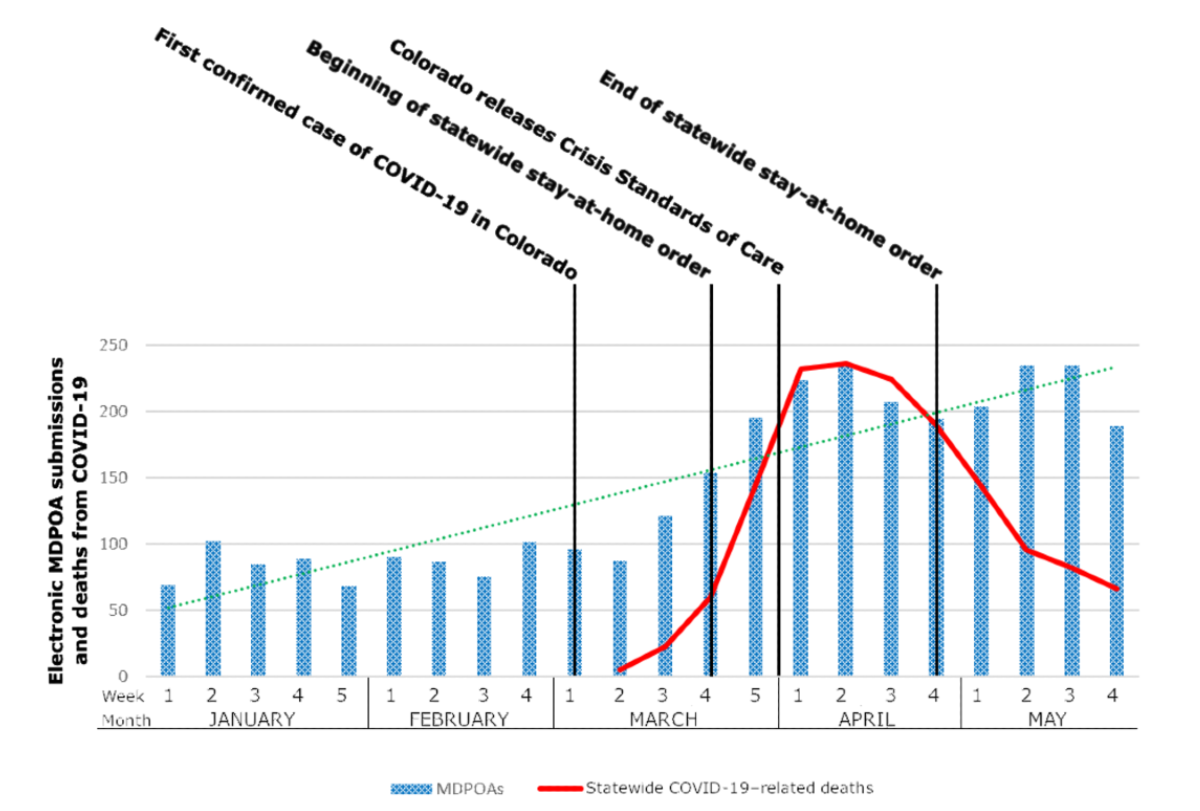
Weekly use of advance care planning portal for electronic MDPOA completion, statewide COVID-19 deaths, and contemporaneous events during the COVID-19 pandemic. COVID-19: coronavirus disease. MDPOA: medical durable power of attorney.

Over a 5-month timeframe that includes the early phase of the COVID-19 pandemic in Colorado, the number of user clicks on the UCHealth advance care planning patient portal page increased from 3511 in January to 6819 in April and 10,077 in May. The total number of monthly advance care planning portal tool users increased from 418 in January to 1037 in April, then slightly decreased to 815 users in May (Figure 2). Week-to-week MDPOA completions varied within each month; however, a positive trend line and greater use were observed in April and May (Figure 3). In an interrupted time-series analysis, the number of MDPOA forms submitted per week increased by 92 after the Colorado stay-at-home order was issued on March 26, 2020 (*P*<.001). The weekly rate was 2.4-fold higher in the 9 weeks after March 26 compared to the nine weeks prior to March 26. Figure 3 shows the number of submissions of MDPOA forms though the UCHealth patient portal in the cultural context of health orders from the State of Colorado. On March 26, 2020, the governor of Colorado issued a statewide stay-at-home policy [26]. On April 5, the government of Colorado approved and publicized Crisis Standards of Care documents as guidelines for how the medical community should allocate scarce resources such as ventilators and intensive care unit beds in the extreme case when patient needs exceed the resources available [27]. The Colorado Department of Public Health and Environment also updated the statewide numbers of cases and deaths of individuals with COVID-19 throughout this time period [28].

From December 29, 2019, to May 30, 2020, 3292 patients used the advance care planning portal tools. These patients were mostly female (2206/3292, 67.0%) with a mean age of 47.7 years (SD 16.1). The ages of patients who used the advance care planning portal tools were relatively evenly distributed between 25 years and ≥65 years (Table 1). The largest group of users was people aged 25-34 years (721/3292, 21.9%). The age distribution in our total population differed significantly between women and men (*P*<.001), where the proportion of women who used the advance care planning portal tools was greater than that of men for all age groups and significantly greater in the younger age groups (Table 2). Regionally, all three health care regions of UCHealth were represented, with 1412/3292 (42.9%) from the metro Denver region.

**Table 1:** Characteristics of patient portal users from December 29, 2019 to May 30, 2020 (N=3292).

Characteristic			n (%)
**Age (years)**			
	18-24	148 (4.5)	
	25-34	721 (21.9)	
	35-44	684 (20.8)	
	45-54	578 (17.6)	
	55-64	543 (16.5)	
	≥65	618 (18.8)	
**Sex**			
	Female	2206 (67.0)	
	Male	1082 (33.2)	
	Nonbinary	4 (0.1)	
**Region**			
	Metro Denver	1412 (42.9)	
	North Colorado	1021 (31.0)	
	South Colorado	719 (21.8)	
	Out of state	140 (4.3)	
**Type of interaction**			
	eMDPOA^a^ completion	3138 (95.3)	
	Message to advance care planning support team	148 (4.5)	
	Invalid MDPOA^b^ submission	6 (0.2)	
**eMDPOA subgroups^c^**			
	No prior agent (oral or MDPOA) and eMDPOA selects a new agent	1665 (50.6)	
	Prior MDPOA form and eMDPOA does not change the agent	644 (19.6)	
	Prior MDPOA from and eMDPOA selects a new agent	78 (2.4)	
	Prior orally appointed agent and eMDPOA does not change the agent	686 (20.8)	
	Prior orally appointed agent and eMDPOA selects a new agent	65 (1.3)	

^a^eMDPOA: electronic medical durable power of attorney.

^b^MDPOA: medical durable power of attorney.

^c^Percentages total 95.3% (proportion of total eMDPOAs).

**Table 2:** Use of the advance care planning patient portal tools by age ( *P*<.001) and gender (male/female).

Age group (years)	Female, n (%)	Male, n (%)
18-24 (n=148)	124 (83.8)	24 (16.2)
25-34 (n=720)	569 (79.0)	151 (21.0)
35-44 (n=682)	465 (68.2)	217 (31.8)
45-54 (n=578)	381 (65.9)	197 (34.1)
55-64 (n=542)	334 (61.6)	208 (38.4)
≥65 years (n=618)	333 (53.9)	285 (46.1)

The primary use of the advance care planning portal tools was the completion of a MDPOA (Table 1). Of the 3292 patients who completed an MDPOA, over 50% (1665/3292, 50.6%) did not have a prior agent in the EHR; thus, these 1665 patients named a medical decision maker during their completion of the MDPOA, ensuring that this information would be available to their health care providers. An additional 686/3292 patients (20.8%) officially appointed a previously orally appointed person to be their legal medical power of attorney by submitting the MDPOA form. The third largest group of patient users (644/3292, 19.6%) were patients who already had an MDPOA on file and did not change their primary medical power of attorney in the new MDPOA, although in some cases they changed an alternate decision maker or added specific instructions as part of the form. Six patients in the 5-month period submitted an invalid MDPOA by selecting themselves as the decision maker or lacked decision-making capacity as determined through quality assurance review and discussion with the primary care provider or emergency contact.

Fewer than 5% of patient portal users who used the advance care planning tools sent an electronic message to a centralized advance care planning support team (148/3292, 4.5%). The majority of these messages were questions related to how users could submit existing paper advance directives so that these directives would be available in the EHR (this feature is planned but not currently available).

## Discussion

### Principal Findings

To our knowledge, this is the first study to identify a significant increase in patient-initiated completion of electronic MDPOAs through a patient portal during the COVID-19 pandemic. In the context of COVID-19, conducting advance care planning is increasingly important to ensure that patients have identified medical decision makers whom they trust and that they receive treatment that aligns with their values and preferences. Patient portals are a particularly useful tool for advance care planning during a pandemic because they are accessible 24 hours per day, do not require face-to-face contact, and are directly linked to health care providers and the EHR.

A significant increase in advance care planning portal tool use was seen in April 2020, aligned with increased awareness of the surge in COVID-19 cases, hospitalizations, and deaths in Colorado, the State of Colorado’s stay-at-home order (March 26 to April 26, 2020), and the widely publicized authorization of the state’s Crisis Standards of Care on April 5. The trend in increased MDPOA completion began in March, which is closely aligned with the announcement of the first COVID-19 case in Colorado (March 5, 2020). Although advance care planning is currently taking place in emergency departments and ICUs [8], these portal users did not submit the MDPOA during an inpatient encounter (data not shown). These users are likely accessing their patient portal account and documenting their preferences at home, away from the clinic and prior to possible emergency care. An existing MDPOA can streamline and enhance the quality of communication during a time of potential health system strain.

Approximately half of the MDPOA forms submitted during this timeframe were new submissions, suggesting that in the setting of the COVID-19 pandemic, people are interested in advance care planning and documenting their health decision makers. COVID-19 has heightened patients’ awareness of the potential of becoming seriously ill. These findings show that patients are willing to act on this knowledge without marked investment in health system resources. While health care providers are calling for improved advance care planning during the pandemic, little is known about patient needs and preferences regarding COVID-19–specific advance care planning processes. The technology of the portal may be an important facilitator. An electronic MDPOA can overcome barriers related to lack of documentation access, lack of ability to share completed forms with the health care system, and perceived time, financial, or legal barriers. These users may welcome the ability to complete the MDPOA through the patient portal instead of with an attorney or health care practitioner because of the need for dedicated appointments, which are likely difficult to access due to stay-at-home orders and clinic preference for telehealth appointments.

During a recent qualitative advance care planning patient portal study, older patients and their caregivers indicated a need for easy access to their current active MDPOA via the portal [29]. Access to the MDPOA enables ongoing review and updating of patient preferences, which are important aspects of advance care planning as an ongoing process [4,30,31]. Patients did use the advance care planning message tool, albeit less frequently; over half of the messages during the study period were sent in the month of April. This corresponds with the MDPOA completion spike in April and indicates that people who are interested in using the portal for advance care planning want to use the portal to share existing advance directives or may need technical assistance to use the portal. Patients have previously reported the need for technical assistance in using advance care planning portal tools [29].

Patients across the age spectrum used the advance care planning portal tools. Surprisingly, the largest age group of the users was 25 to 34 years, demonstrating that users in this age group are interested in engaging in advance care planning by choosing a medical decision maker. Advance care planning typically increases with age [32,33]. In Colorado, only 17% of adults aged 25 to 34 years have an advance directive, compared to 66% of adults over the age of 65 [25]. Although it is recommended to engage young adults in advance care planning, the majority of efforts target older adults or focus on young adults with life-limiting illness. Lack of advance care planning awareness and perception among healthy young adults that advance care planning is unnecessary are two major barriers to their involvement in advance care planning. However, young adults who have previous experiences with a seriously ill loved one have demonstrated increased advance care planning awareness and preference for completing an advance directive [30,31]. Contextually, at the end of May 2020, approximately 33% of Coloradans diagnosed with COVID-19 were aged 20 to 39 years (approximately 8728 individuals, as reported on June 6, 2020), and 6.6% of these cases resulted in hospitalization [28]. Because COVID-19 does not only affect older adults, young people may see the value in advance care planning. The patient portal mechanism for advance care planning documentation aligned with health communication and health information access values patients in the younger age demographic; therefore, patient portals are a sustaining strategy for promoting advance care planning in this population [34].

In addition to advance care planning, age is associated with patient portal adoption and use [35,36]. Healthy young adults and older adults, particularly those over the age of 75, are less likely to register for a patient portal account and to regularly use portal features. While this lack of portal use among younger adults is typically associated with their health status, lack of portal use by older adults is attributed to a lack of technology access or technical barriers [37]. Our studies indicate that patients chose to use the portal for advance care planning regardless of age and technical barriers. However, efforts should be made to increase advance care planning awareness among younger adults and address technical concerns among older adults.

In this cohort, women were more likely to complete an MDPOA across all age groups; however, this difference was more pronounced in younger age groups. Gender differences have been found in both patient portal use and advance care planning, with women being more likely to engage with a portal [36] and with advance care planning [25,32,33]. Even among young adults, a national survey found that male respondents reported less familiarity with the concepts of advance care planning, advance directives, and health care proxies [30]. In Colorado, approximately 48% of patients with COVID-19 are male; however, male patients represent 56% of COVID-19 deaths [28].

### Limitations

There are limitations to our study. Our data lacked information on race, ethnicity, education status, and specific geographic location (ie, zip code) to determine rurality, which are all known to impact access to health care, health technology use, and advance care planning engagement [32]. We also did not capture the health status, medical diagnoses, prior engagement with advance care planning, or overall preferences related to consumer health technology, which can also be associated with patient portal use. While our study identified a significant increase in engagement with the portal over time, due to the retrospective cohort design of our study, we are unable to conclude that this increase is solely caused by the COVID-19 pandemic rather than other secular trends. Also, it is difficult to predict if the observed growth will be sustained throughout the pandemic period or if there was an immediate effect during the stay-at-home order. As seen in Figure 2, there was a slight decrease in portal use in May; therefore, ongoing investigation of the rate of engagement via the portal is warranted, as attention to advance care planning is beneficial as COVID-19 continues to spread across the United States. However, the use of electronic MDPOAs is not feasible in all states due to state-level requirements; this limits generalizability across states, where state laws may preclude use of the portal for completion of a legal MDPOA. Future research is needed to assess these potential covariates and capture user experiences regarding advance care planning portal use. Research should explore possible associations between patient MDPOA completion and the care provided in the treatment of COVID-19.

### Conclusion

The use of a patient portal to complete a MDPOA form to choose a legal medical decision maker increased substantially during the first months of the spread of COVID-19 in the state of Colorado. With the currently increased interest in advance care planning among patients and health care providers, patient portals may be an important tool for advance care planning during the COVID-19 pandemic to enhance and supplement face-to-face advance care planning efforts in health systems.

## Abbreviations

COVID-19: coronavirus disease
EHR: electronic health record
ICU: intensive care unit
MDPOA: medical durable power of attorney
SARS-CoV-2: severe acute respiratory syndrome coronavirus 2

## References

[1] (2020). Report of the WHO-China Joint Mission on Coronavirus Disease 2019 (COVID-19). World Health Organization.

[2] Stokes EK, Zambrano LD, Anderson KN, Marder EP, Raz KM, El Burai Felix S, Tie Y, Fullerton KE (2020). Coronavirus Disease 2019 Case Surveillance - United States, January 22-May 30, 2020. MMWR Morb Mortal Wkly Rep.

[3] Advance Care Planning. National Hospice and Palliative Care Organization.

[4] Sudore RL, Lum HD, You JJ, Hanson LC, Meier DE, Pantilat SZ, Matlock DD, Rietjens JAC, Korfage IJ, Ritchie CS, Kutner JS, Teno JM, Thomas J, McMahan RD, Heyland DK (2017). Defining Advance Care Planning for Adults: A Consensus Definition From a Multidisciplinary Delphi Panel. J Pain Symptom Manage.

[5] Advance Care Planning: Healthcare Directives. National Institute on Aging.

[6] Brinkman-Stoppelenburg A, Rietjens JAC, van der Heide A (2014). The effects of advance care planning on end-of-life care: a systematic review. Palliat Med.

[7] Houben CHM, Spruit MA, Groenen MTJ, Wouters EFM, Janssen DJA (2014). Efficacy of advance care planning: a systematic review and meta-analysis. J Am Med Dir Assoc.

[8] Curtis JR, Kross EK, Stapleton RD (2020). The Importance of Addressing Advance Care Planning and Decisions About Do-Not-Resuscitate Orders During Novel Coronavirus 2019 (COVID-19). JAMA.

[9] (2017). Telemedicine and Telehealth. Office of the National Coordinator for Health Information Technology (ONC).

[10] Padala PR, Jendro AM, Gauss CH, Orr LC, Dean KT, Wilson KB, Parkes CM, Padala KP (2020). Participant and Caregiver Perspectives on Clinical Research During Covid-19 Pandemic. J Am Geriatr Soc.

[11] Dewar S, Lee PG, Suh TT, Min L (2020). Uptake of Virtual Visits in A Geriatric Primary Care Clinic During the COVID-19 Pandemic. J Am Geriatr Soc.

[12] Hong Y, Lawrence J, Williams D, Mainous I (2020). Population-Level Interest and Telehealth Capacity of US Hospitals in Response to COVID-19: Cross-Sectional Analysis of Google Search and National Hospital Survey Data. JMIR Public Health Surveill.

[13] Mahmood S, Hasan K, Colder Carras M, Labrique A (2020). Global Preparedness Against COVID-19: We Must Leverage the Power of Digital Health. JMIR Public Health Surveill.

[14] Ohannessian R, Duong TA, Odone A (2020). Global Telemedicine Implementation and Integration Within Health Systems to Fight the COVID-19 Pandemic: A Call to Action. JMIR Public Health Surveill.

[15] van der Smissen D, Overbeek A, van Dulmen S, van Gemert-Pijnen L, van der Heide A, Rietjens JA, Korfage IJ (2020). The Feasibility and Effectiveness of Web-Based Advance Care Planning Programs: Scoping Review. J Med Internet Res.

[16] Goldzweig CL, Orshansky G, Paige NM, Towfigh AA, Haggstrom DA, Miake-Lye I, Beroes JM, Shekelle PG (2013). Electronic patient portals: evidence on health outcomes, satisfaction, efficiency, and attitudes: a systematic review. Ann Intern Med.

[17] Bose-Brill S, Pressler TR (2012). Commentary: opportunities for innovation and improvement in advance care planning using a tethered patient portal in the electronic health record. J Prim Care Community Health.

[18] Vydra TP, Cuaresma E, Kretovics M, Bose-Brill S (2015). Diffusion and Use of Tethered Personal Health Records in Primary Care. Perspect Health Inf Manag.

[19] Lum HD, Sudore RL, Matlock DD, Juarez-Colunga E, Jones J, Nowels M, Schwartz RS, Kutner JS, Levy CR (2017). A Group Visit Initiative Improves Advance Care Planning Documentation among Older Adults in Primary Care. J Am Board Fam Med.

[20] Bose-Brill S, Feeney M, Prater L, Miles L, Corbett A, Koesters S (2018). Validation of a Novel Electronic Health Record Patient Portal Advance Care Planning Delivery System. J Med Internet Res.

[21] Brungardt A, Daddato AE, Parnes B, Lum HD (2019). Use of an Ambulatory Patient Portal for Advance Care Planning Engagement. J Am Board Fam Med.

[22] Jordan SR, Brungardt A, Phimphasone-Brady P, Lum HD (2019). Patient Perspectives on Advance Care Planning via a Patient Portal. Am J Hosp Palliat Care.

[23] Lum HD, Brungardt A, Jordan SR, Phimphasone-Brady P, Schilling LM, Lin C, Kutner JS (2019). Design and Implementation of Patient Portal-Based Advance Care Planning Tools. J Pain Symptom Manage.

[24] Walling AM, Sudore RL, Bell D, Tseng C, Ritchie C, Hays RD, Gibbs L, Rahimi M, Sanz J, Wenger NS (2019). Population-Based Pragmatic Trial of Advance Care Planning in Primary Care in the University of California Health System. J Palliat Med.

[25] (2018). Advance Care Planning in Colorado. Colorado Health Institute.

[26] State at Home Order. Colorado Department of Public Health and Environment.

[27] Colorado Crisis Standards of Care. Colorado Department of Public Health & Environment.

[28] COVID-19 Case Data. Colorado Department of Public Health & Environment.

[29] Portz JD, Lum HD, Bull S, Boxer RS, Bekelman DB, Ford KL, Gleason K, Casillas A, Bayliss EA (2020). Perceptions of Patient Portal Use for Advance Directive Documentation among Older Adults with Multiple Chronic Conditions. J Soc Work End Life Palliat Care.

[30] Tripken JL, Elrod CS (2018). Young Adults' Perspectives on Advance Care Planning. Am J Hosp Palliat Care.

[31] Kavalieratos D, Ernecoff NC, Keim-Malpass J, Degenholtz HB (2015). Knowledge, attitudes, and preferences of healthy young adults regarding advance care planning: a focus group study of university students in Pittsburgh, USA. BMC Public Health.

[32] Bischoff KE, Sudore R, Miao Y, Boscardin WJ, Smith AK (2013). Advance care planning and the quality of end-of-life care in older adults. J Am Geriatr Soc.

[33] Sable-Smith A, Arnett KR, Nowels MA, Colborn K, Lum HD, Nowels D (2018). Interactions with the healthcare system influence advance care planning activities: results from a representative survey in 11 developed countries. Fam Pract.

[34] Peacock S, Reddy A, Leveille SG, Walker J, Payne TH, Oster NV, Elmore JG (2017). Patient portals and personal health information online: perception, access, and use by US adults. J Am Med Inform Assoc.

[35] Ancker JS, Osorio SN, Cheriff A, Cole CL, Silver M, Kaushal R (2015). Patient activation and use of an electronic patient portal. Inform Health Soc Care.

[36] Irizarry T, DeVito Dabbs A, Curran CR (2015). Patient Portals and Patient Engagement: A State of the Science Review. J Med Internet Res.

[37] Gordon NP, Hornbrook MC (2016). Differences in Access to and Preferences for Using Patient Portals and Other eHealth Technologies Based on Race, Ethnicity, and Age: A Database and Survey Study of Seniors in a Large Health Plan. J Med Internet Res.

